# Molecularly Imprinted Polymer Micro- and Nano-Particles: A Review

**DOI:** 10.3390/molecules25204740

**Published:** 2020-10-15

**Authors:** Beatriz Fresco-Cala, Alex D. Batista, Soledad Cárdenas

**Affiliations:** 1 Institute of Analytical and Bioanalytical Chemistry, Ulm University, 89081 Ulm, Germany; alexdbatista@gmail.com; 2 Departamento de Química Analítica, Instituto Universitario de Investigación en Química Fina y Nanoquímica IUNAN, Universidad de Córdoba, Campus de Rabanales, Edificio Marie Curie, E-14071 Córdoba, Spain

**Keywords:** molecularly imprinted polymer, hybrid sorbent, nanomaterial, monolith, microextraction, sensor

## Abstract

In recent years, molecularly imprinted polymers (MIPs) have become an excellent solution to the selective and sensitive determination of target molecules in complex matrices where other similar and relative structural compounds could coexist. Although MIPs show the inherent properties of the polymers, including stability, robustness, and easy/cheap synthesis, some of their characteristics can be enhanced, or new functionalities can be obtained when nanoparticles are incorporated in their polymeric structure. The great variety of nanoparticles available significantly increase the possibility of finding the adequate design of nanostructured MIP for each analytical problem. Moreover, different structures (i.e., monolithic solids or MIPs micro/nanoparticles) can be produced depending on the used synthesis approach. This review aims to summarize and describe the most recent and innovative strategies since 2015, based on the combination of MIPs with nanoparticles. The role of the nanoparticles in the polymerization, as well as in the imprinting and adsorption efficiency, is also discussed through the review.

## 1. Introduction

Molecularly imprinted polymers (MIPs) are selective sorbents for extracting a target molecule, which essentially mimics the ‘lock-and-key’ binding mechanism occurring in natural biorecognition. To achieve this goal, a template molecule, which can be the target compound, a fragment of it, or a molecule with a size, shape, and functional groups similar to the target one (dummy template), is added to the polymerization mixture to interact with the monomers forming a complex [1,2]. Thus, when the propagation of polymeric chains progresses, the template molecule is surrounded and consequently trapped in the three-dimensional polymer network. After extracting with suitable solvents, the template is removed, ideally leaving selective recognizing sites that are complementary to the target species. The resulting MIPs have the inherent advantages of synthetic polymers, such as high mechanical stability in a wide range of solvents, pH, and temperature. These polymers are also very robust even at elevated pressure and offer the possibility of preparing them in different types of formats/supports. Additionally, MIPs show high selectivity and an improved adsorption efficiency in comparison with their analogous prepared without template (molecularly non-imprinted polymer, NIPs). For all this, MIPs are currently being used in a wide of analytical applications, including chromatography [3,4], microextraction [5,6], and sensing [7].

Nanoparticles exhibit exceptional and different properties than macroscopic matter [8]. These unique properties depend mainly on the type of nanoparticle, as well as on their shape and size, which allows the use of one type or another according to the specific analytical need. This has opened a new window of possibilities for developing new analytical methods [9,10,11]. In this way, an innovative way to synthesize novel MIPs is the incorporation of nanoparticles to their structure [12,13]. The combination of these two materials (polymer and nanoparticles) gives rise to a hybrid material with potential and new properties.

In general, MIPs are classified into two types according to whether they are obtained as a single continuous and porous piece (molecularly-imprinted monoliths, MIMs) [14] or as individual nano/microparticles (MIP micro/nanoparticles) [15,16]. When MIMs are prepared, nanoparticles can be directly dispersed in the porogen solvents, and after the polymerization, a hybrid solid with embedded nanoparticles is obtained. Some nanoparticles, such as conical carbon nanoparticles or molecular sieves, can be acted as main monomers or scaffolds of the monolithic structure. In contrast, in the synthesis of MIP nanoparticles, the role of nanoparticles, generally, is to act as the core or support of the imprinted polymer film. While solvents and sample solutions flow through the pores of the MIMs, normally thanks to the help of an external force (i.e., micro-HPLC or syringe pump, centrifugation, or vacuum pump), MIP nanoparticles are dispersed in the samples, and once the extraction performance is finished, they are recovered.

This review summarizes the most recent analytical applications that use both MIMs and MIP nanoparticles. Different synthetic routes to obtain nanostructured MIPs are described emphasizing the role of nanoparticles during the polymerization stage, as well as their role during extraction/detection of the target analyte.

## 2. Types of Strategies to Synthesize Nanostructured MIPs

In general, MIPs can be prepared via different polymerization strategies [3], including bulk imprinting (3D) and surface imprinting (2D). In surface imprinting, templates are located at the surface of the material, while in bulk imprinting, monolithic solids are generated with the template embedded into the cross-linked structure. Especially for the fabrication of MIPs for (bio)macromolecules, surface imprinting remains advantageous as diffusion to and binding of template/analyte at the polymer surface is more favorable and faster than inside its porous structure. The most commonly applied surface imprinting strategies include soft lithography [17,18], self-assembled monolayers [19], core-shell particles preparation [20,21], and miniemulsion polymerization [22,23].

For adequately designing the recognizing sites in a MIP, different synthetic strategies in terms of the interactions between functional monomers and the template molecules are currently used. The non-covalent imprinting approach pioneered by Mosbach et al. [24] is based on the interactions of the template with the functional monomers during the polymerization step through weak forces, i.e., Van der Waals forces, hydrogen bonding, or π-π interactions, which results in facile template removal. Also, selective recognition during the analyte binding step relies on non-covalent interactions. Non-covalent imprinting is a simple and effective approach, and although it has some issues related to relatively easy disruptions of these interactions during the formation of the template-monomer complex, it remains the most commonly used strategy to date [25,26]. While a wide variety of functional monomers, including acid, basic and neutral monomers (e.g., acrylamide (AM) [27], 2-hydroxyethyl methacrylate [28], vinylimidazole [29], aminostyrene [30], methacrylic [31], and acrylic [32] acid), can be applied in non-covalent imprinting, only functional monomers with the capacity to form reversible condensation reactions with the template (e.g., boronic esters [33] and Schiff bases [34] can be used in a covalent approach. Besides, slower binding and a more tedious template removal (i.e., covalent bond cleavage) are associated with the covalent imprinting strategy. However, whi9te it has been demonstrated that strong non-covalent stoichiometric interactions can also provide oriented and homogeneously distributed molecularly imprinted cavities [35], the covalent approach remains the most widely used at present to get to assign and fix the orientation of the interacting recognizing sites. To overcome the limitations of both approaches, a semi-covalent approach was devised [36]. This intermediate approach involves covalent binding between the template and functional monomer during the preparation of the MIP and the subsequent non-covalent interactions during the analyte binding in the application. Hence, semi-covalent imprinting combines the advantages of covalent and non-covalent approaches [37,38].

Precipitation polymerization and emulsion polymerization are the most used polymerization methods to synthesize MIP NPs [7,15,16], while bulk polymerization has traditionally been used to synthesize MIPs as porous monoliths [14,39]. In both cases, the polymerization reaction can be initiated using thermal and UV energy in the presence of a radical initiator (e.g., methacrylate and acrylate polymers) [40,41] or thanks to a change of the pH value in polycondensation reactions (e.g., silica polymer) [42,43]. On the other hand, electropolymerization is widely used for the preparation of thin polymeric layers [44,45].

## 3. Analytical Applications of MIMs with Nanoparticles

MIMs have been widely used for analytical purposes, both in-sample pretreatment and chromatographic separation [14]. They combine the advantages of MIPs and monolithic solids, among which stand out their versatility and ease synthesis in many formats, their low cost, and their high selectivity. MIMs can also be synthesized in the presence of nanoparticles, which would form part of the final selective sorbent. However, the nanoparticles can be only embedded in the polymeric network, increasing the surface area of the material or providing the sorbent an extra/new property, such as magnetism in the case of incorporating magnetic nanoparticles or having a crucial role in the formation of the monolithic structure, being themselves the scaffolding. Thus, the following sections describe the analytical applications of this type of hybrid imprinted sorbents in recent years according to the role of nanoparticles in the preparation and characteristics of the MIMs. Some review articles and book chapters had also discussed the use of micro and nanoparticles with MIPs for different applications [12,46,47,48,49].

### 3.1. MIMs with Embedded Nanoparticles

In 2015, MIPs coated graphene oxide (GO) were polymerized in situ inside monolithic capillary columns for their use as extraction units coupled with HPLC-LIF [50]. Phloxine B was selected as a target analyte, and it was also used as a template during the imprinting. GO was firstly synthesized by the method of Hummer and Offeman [51], and then, GO was used as supporting material for the formation of the MIP. For that, the functional monomer (methacrylic acid, MAA), the cross-linking monomer (ethylene glycol dimethylacrylate, EGDMA), the template (Phloxine B), and the GO were mixed, and the polymerization reaction was initiated by adding the radical initiator (azobisisobutyronitrile, AIBN) and carried out at 60 °C for 5 h. AM was also tested as a functional monomer, however, monoliths with gaps and an unshapely structure were formed in comparison with the uniform and continuous monoliths prepared with MAA. The incorporation of GO in the monolithic structure improved the extraction capacity of the column, probably due to the increase of the specific surface area of the material. In this way, the extraction efficiency toward Phloxine B achieved with the GO-MIP was higher than the obtained both with the NIP and the MIP prepared without GO. The selectivity of the GO-MIP column was also corroborated by the calculation of the column capacity for the Phloxine B (0.040 mg·mg^−1^), as well as for rose bengal (0.020 mg·mg^−1^)—a compound structurally similar to phloxine B. Finally, the GO-MIP was applied to extract Phloxine B in coffee bean samples obtaining recovery between 89.5 and 91.4% and a LOD of 0.075 ng·mL^−1^. Quantum dots (QDs) were also embedded in a MIM, which was synthesized directly in a capillary column by thermal polymerization (55 °C, 6 h) [52]. In this case, a dummy template (5,7-dimethoxycoumarin, DMC) was used for the formation of selective cavities for aflatoxin B1—mycotoxin produced by *Aspergillus flavus* and *A. parasiticus*—due to the high toxicity and cost of the aflatoxin B1. The composition of the polymerization mixture (monomers and porogens) was optimized to achieve a high imprinting efficiency, as well as obtain a robust and uniform solid. Moreover, polymerization was attempted under UV radiation, but the polymerization reaction was incomplete. The effect of the presence of the QDs was also studied synthesizing monolithic columns with and without them. The pores formed when QDs were in the polymerization mixture were more uniform, and therefore, these columns showed higher extraction capacity with an enrichment factor over 71-fold. Additionally, the pressure generated when the solvents and the sample solutions flowed through the column was lower. QD-dummy molecularly imprinted polymer columns were used to the isolation and preconcentration of aflatoxin B1 in peanut samples (the recovery ranged from 79.5 to 91.2%), while the final extract containing the target analyte was analyzed by HPLC-FLD. The LOD of the method was 0.118 ng·mL^−1^.

Monolithic solids containing magnetic nanoparticles (MNPs) can be used as stir bars without the need of prepared them over any support [53,54]. The continuous and porous structure of the monoliths, together with the magnetic response of the MNPs, offers the possibility of obtaining hybrid and robust macroscopic bars able to rotate in the sample solution in the presence of an external magnetic force. In this way, a monolithic stir bar based on propazine-MIP for the determination of triazines in environmental soil samples was fabricated [55]. To prevent the degradation of the MNPs during polymerization, they were functionalized with oleic acid and then covered with a silica layer. Moreover, the silica surface was modified with vinyl groups to improve the interactions with the monomers. This study revealed that the incorporation of the MNPs negatively affected the polymerization, needing more time to complete the reaction when the high content of nanoparticles (28 wt%) was added to the mixture. On the other hand, the low content of MNPs (<7 wt%) did not allow the rotation of the monolithic bar. Therefore, optimum monolithic stir bars were prepared with the content of nanoparticles between 7 and 14 wt% of MNPs, and they were used for more than 30 times without extraction efficiency losses. The quantification was carried out by HPLC-UV, and the LODs of the method were in the range of 3.6–7.5 ng·g^−1^. The same group prepared thiabendazole (TBZ) imprinted stir bar introducing modified-MNPs in the monolithic network again [56]. In this case, the monolithic stir bars were applied to determine TBZ and carbendazim (CBZ) in orange samples, being the LODs 0.1 and 0.13 mg·kg^−1^, respectively.

### 3.2. MIMs Formed Mainly by Nanoparticles

**Scheme 1:**
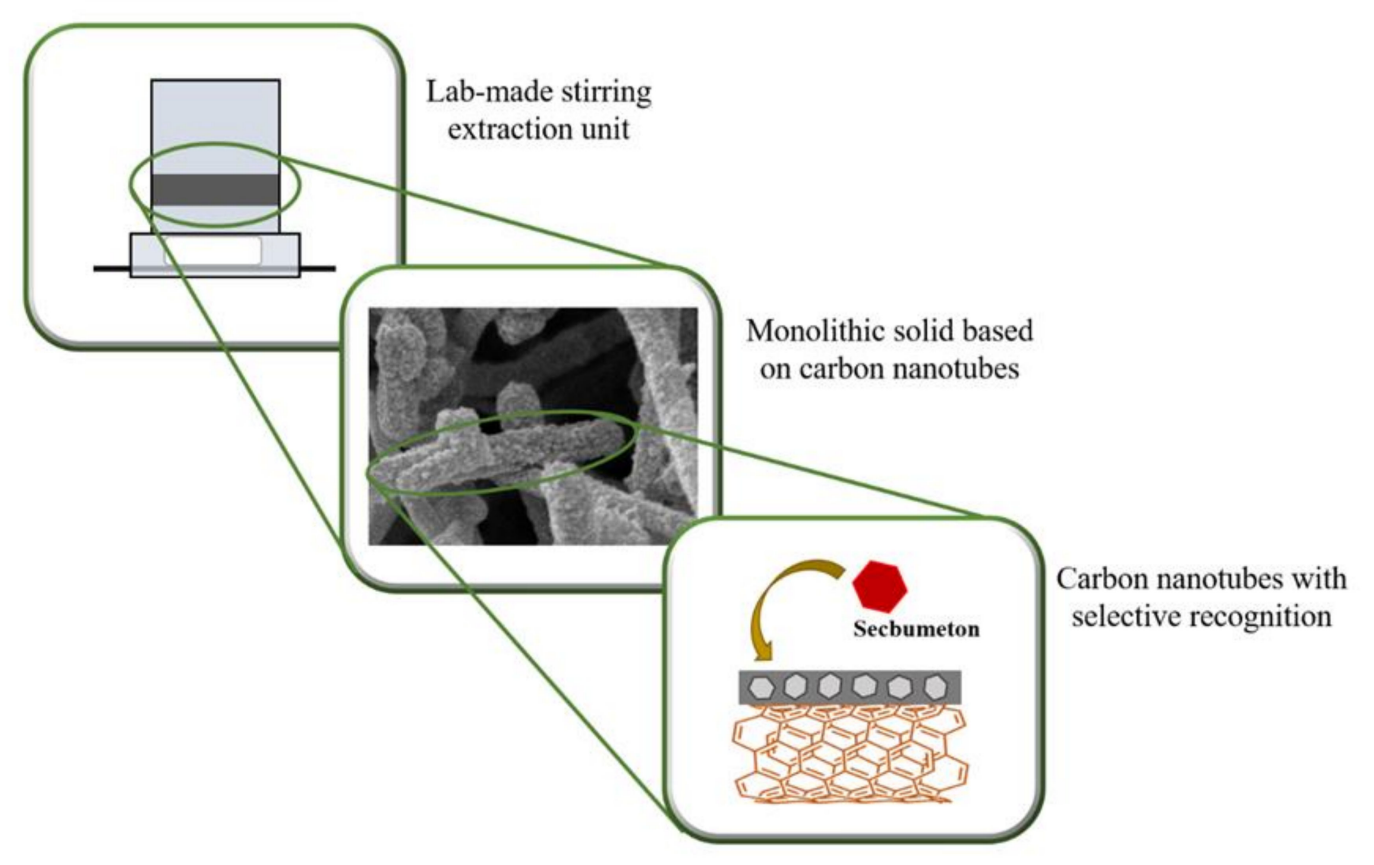
Main elements of the molecularly imprinted polymer stirring unit synthesized by interconnected carbon nanotube monolithic sorbent in a polypropylene cap using secbumenton as a template. Reproduced with permission from [60].

As an alternative to monolithic solids with nanoparticles embedded in their polymeric structure, there is the possibility of interconnecting the individual nanoparticles to form a single and continuous network. Thus, the nanoparticles are generally linked to each other by a thin polymer coating (cross-linker) to give rise to a porous monolithic solid [57,58,59]. In this way, a monolithic solid based on interconnected carbon nanotubes via the formation of a W/O medium internal phase emulsion followed by a photopolymerization approach was prepared [60]. On the one hand, the aqueous phase, which was inside the droplets formed by the surfactant (L121), provided the pores to the final structure, while the polymerization reaction took place in the oil phase. Carbon nanotubes were trapped in the oil phase, due to their hydrophobicity and a thin layer of EGDMA and MAA covered the surface of the carbon nanotubes to facilitate the formation of the 3D structure. With the addition of a template (secbumeton) to the polymerization mixture, selective cavities were created in the thin polymeric layer (Scheme 1). As can be seen in Scheme 1, the polymerization was directly carried out in a pretreated lab-made stirring extraction unit. This stirring unit was fabricated using a polypropylene tube and ironware, which allow the system rotation. Additionally, the upper of the unit (above the monolithic solid) acted as a container for the eluent, which simplified and significantly improved the elution step. Thus, these innovative monolithic stirring extraction units were applied to determine triazine herbicides in peppermint mint and tea samples. GC-MS was selected for the quantitative analysis, and the LODs were to between 0.4 and 2.5 μg·L^−1^. The recovery ranged from 74 to 122%, with an RSD lower than 13% in all cases.

Nanoparticles can also be used as mesoporous molecular sieve scaffolds to synthesize the MIP [61]. In this work, MCM-41—nanosilica material with large pore size and high surface area—was used as a dendritic scaffold to synthesize MIM containing polyhedral oligomeric silsesquioxanes (POSS). For this purpose, a POSS monomer (1-propylmethacrylateheptaisobutyl substituted), a cross-linking monomer (EGDMA), a functional monomer (4-vinylpyridine, 4-VP), and a template (s-naproxen) were polymerized at 60 °C for 3 h in a stainless-steel column. The presence of the POSS improved the formation of recognizing sites, and therefore, a more selective recognition ability and a high adsorption performance of MIPs were obtained. The specific surface area was increased with the increase of the amount of MCM-41, although the introduction of high amounts of it (≥30.6 mg) caused aggregates and precipitate in the polymerization mixture, and consequently, the resulting monoliths were not available for the pass of the sample solution. Noticeably, the monolithic columns prepared with MCM-41 nanoparticles and POSS monomers showed a higher imprinting effect, as well as higher recovery of s-naproxen than the s-naproxen-MIPs synthesized in the absence of them.

## 4. Analytical Applications of MIP Nanoparticles

MIP nanoparticles represented an important advance in imprinting technology, as they overcome several drawbacks of the MIP obtained by bulk polymerization, such as the unequal distribution of the recognizing sites, irregular morphology, incomplete template removal, and slow mass transfers [62]. Despite the more complex synthesis of the MIP nanoparticles, the mentioned advantages compensate for their laborious synthesis procedures.

Nanoparticles are employed as matrix support for a MIP shell and must be synthesized according to the physical and chemical properties of the template and functional monomers employed for the MIP shell synthesis. The nanoparticles that act as the MIP core usually require some type of surface modification to provide new properties to ensure the effective polymerization of the MIP onto their surfaces [7]. Nanoparticles can also be employed to improve the physical or chemical properties of the MIP for detectability enhancement. In this section, the recent use of polymeric, silica, carbon, and AuNPs for the fabrication of MIP is presented.

### 4.1. Polymeric Nanoparticles

Molecularly imprinted nanoparticles, MIP nanoparticles (NPs), or nanoMIPs, with molecular cavities featuring selective recognizing sites, have widely used for the extraction and preconcentration of both small- and macromolecules from complex samples [63,64,65]. The precipitation polymerization method is one of the most popular ways to obtain uniform imprinted polymeric nanoparticles to retain small molecules, such as triazine herbicides [66,67]. For instance, symetrin-imprinted nanoparticles have been synthesized with this method using MAA and EGDMA as functional monomer and cross-linker, respectively, in the presence of the template (symetrin) [67]. The functional monomer was selected based on the results of the electrostatic potential distribution simulation during the formation of simetryn-monomer complex. On the contrary, the synthesis conditions, including reaction time, solvent volume, as well as the need to stir the polymerization mixture during the preparation step, were experimentally evaluated to find the optimal values. Once MIP nanoparticles have been polymerized and washed, they were packed in an SPE cartridge, and the high selectivity of the prepared nanoparticles was demonstrated by the successful extraction of four triazines from tobacco samples and their subsequent quantification/detection in UHPLC-MS/MS. The LODS ranged from 6 to 30 ng·mL^−1^, and the recovery was between 84.03 and 119.05%.

An interesting and innovative alternative to methacrylate-based monomers is the use of deep eutectic solvents (DESs) as functional monomers for the MIP nanoparticle-preparation [68]. For this purpose, the DESs—which consisted of a mixture of caffeic acid (CA), choline chloride (ChCl), and ethylene glycol (EG)—were previously immobilized on the surface of activated hexagonal boron nitride (h-BN) and then was mixed with EGDMA (cross-linking monomer), the template (quercetin), methanol (porogen) and AIBN (initiator). The polymerization reaction was carried out at 60 °C for 12 h. MIP nanoparticles without h-BN were also prepared and characterized, concluding that the introduction of h-BN scaffolds increased the surface of the final nanoparticles. The addition of the template to the polymerization mixture also affected the morphology of the nanoparticles, since larger pore sizes and pore volumes were found when the polymerization was carried out in the presence of the template molecule. The h-BN-MIP nanoparticles were applied for the purification of 3 flavonoids (quercetin, isorhamnetin, and kaempferol) from Ginkgo biloba leaves. SPE procedure execution followed by HPLC-UV determination was used to obtain recovery values between 94.3% (for kaempferol) and 97.6% (for quercetin) and RSDs lower than 1.0%.

**Scheme 2:**
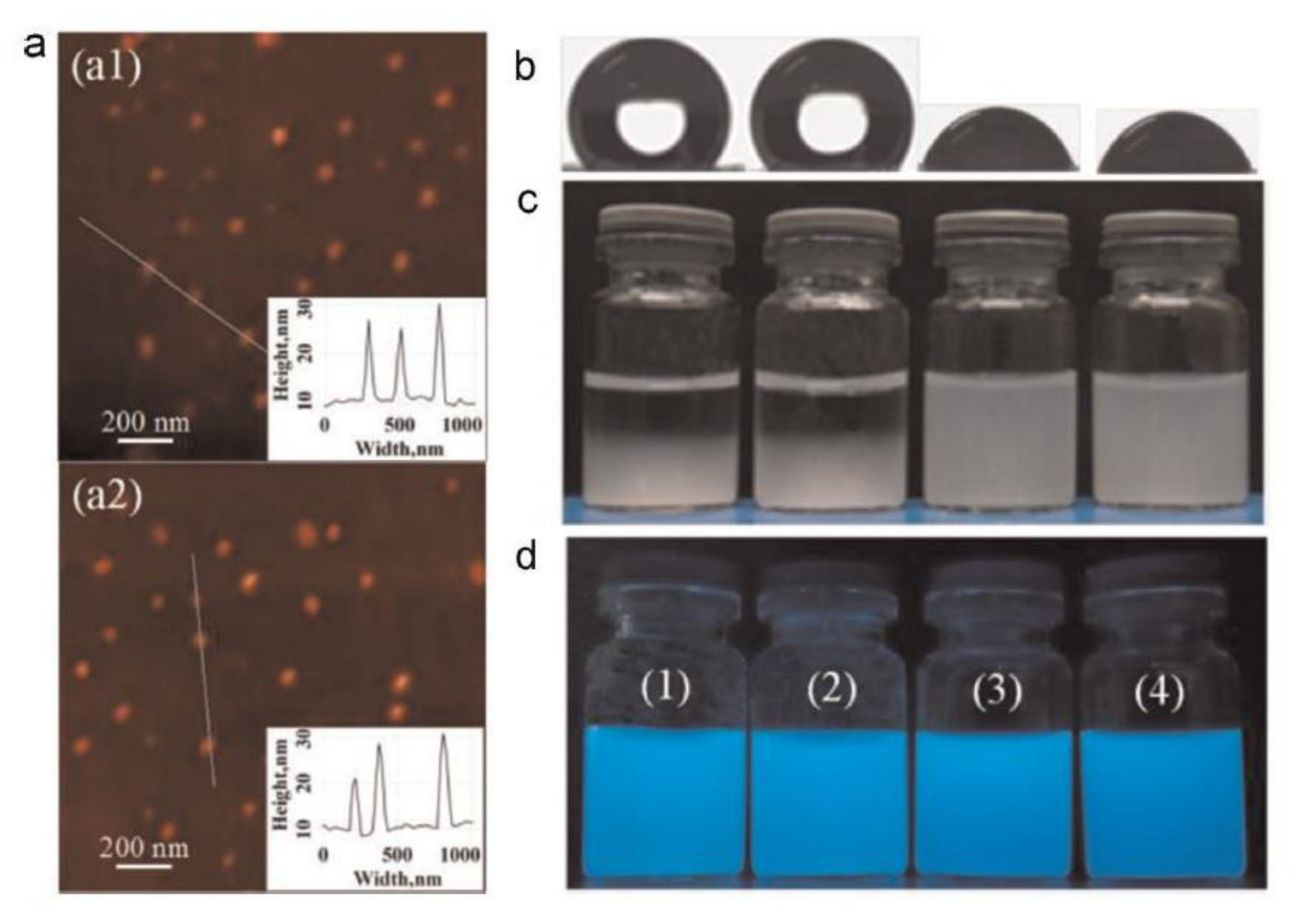
(**a**) AFM images of the grafted fluorescent tetracycline-imprinted polymer (a1)/non-imprinted polymer (a2) nanoparticles. (The inset is a height profile of cross-section (white line)). (**b**) Profiles of a water drop on the films of the ungrafted and grafted fluorescent tetracycline-imprinted polymer/non-imprinted polymer nanoparticles. Photographs of nanoparticles dispersed in pure water (1 mg·mL^−1^) (**c**) without irradiation and (**d**) under 365-nm light irradiation. The samples from left to right in each figure are the ungrafted tetracycline-imprinted polymer (1)/non-imprinted polymer nanoparticles (2) and grafted tetracycline-imprinted polymer (3)/non-imprinted polymer nanoparticles (4). Reproduced with permission from [69].

**Scheme 3:**
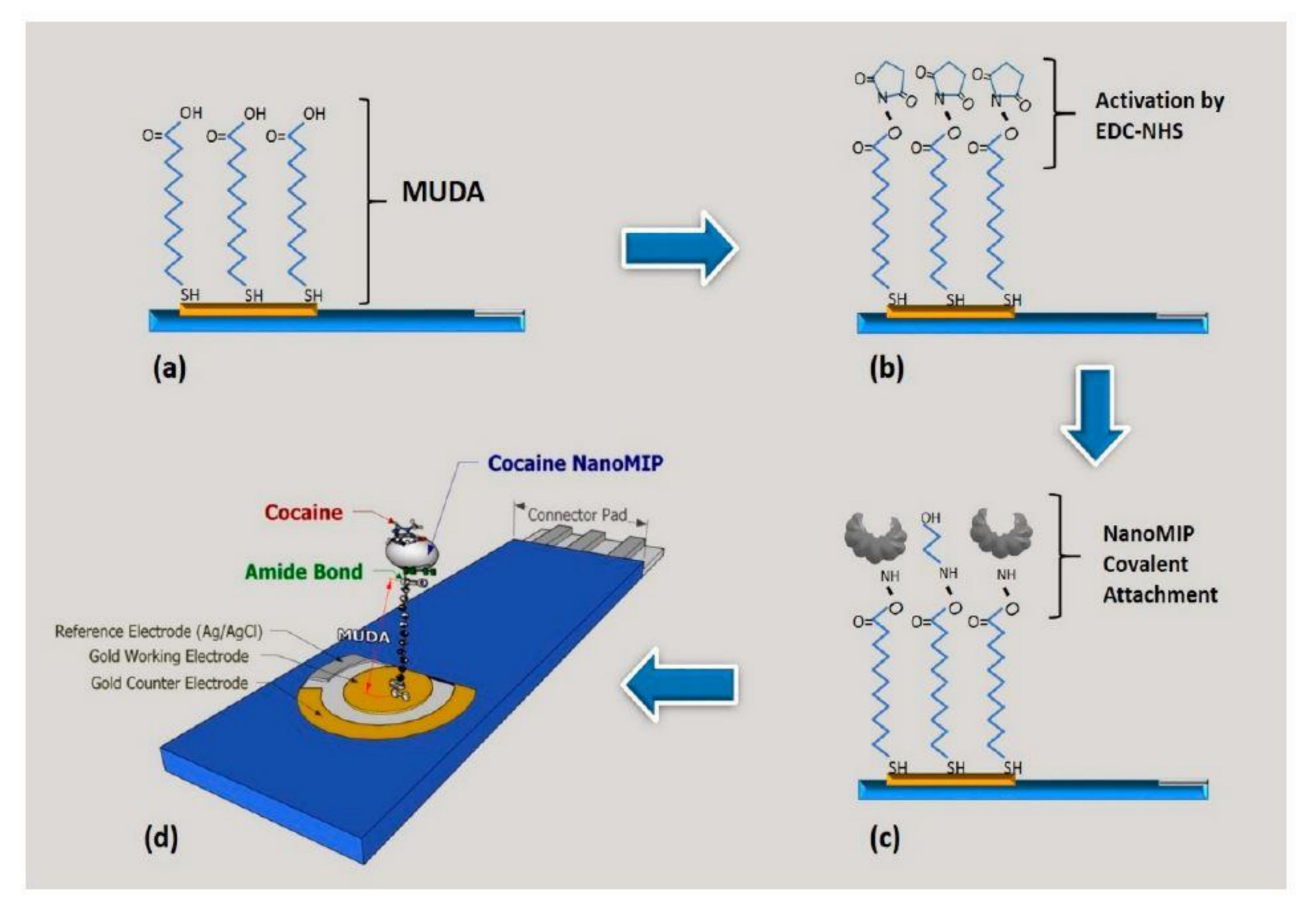
Steps of attaching the nano-molecularly imprinted polymers (nanoMIPs) onto the gold working electrode surface (DPR C220AT, DropSens). (**a**) self-assembly monolayer formation; (**b**) carboxylic group activation by 1-Ethyl-3-(3-dimethylaminopropyl) carbodiimide/Hydroxysuccinimide; (**c**) nanoMIPs covalent attachment via amine coupling. (**d**) 3D scheme of the final nanoMIP sensor for cocaine determination. The scheme not in scale. Reproduced with permission from [79].

The preparation of MIP sensors based on fluorescence properties has attracted increasing attention in the last years [69,70,71]. In 2015, a hydrophilic fluorescent MIP nanoparticle was prepared via a one-pot synthesis approach to determine drugs in biological (bovine and pig serum) samples [69]. The MIP nanoparticles consisted of a fluorescent core, which was formed by a fluorescent monomer (2-hydroxyethylanthrancene-9-carboxylate)methacrylate, AnHEMA), a functional monomer (MAA), a cross-linking monomer (EGDMA), and the template (tetracycline), and a surface grafted with poly(2-hydroxyethyl methacrylate) (poyHEMA) brushes, which gave rise to highly hydrophilic nanoparticles. MIP nanoparticles with and without polyHEMA brushes, as well as their corresponding NIPs were prepared and characterized confirming that the MIP nanoparticles grafted with polyHEMA brushes had significantly smaller diameters (between 10–20 nm, as it can be seen in Scheme 2a) than ungrafted NPs (around 3.4 µm). Besides, the higher hydrophilicity of the grafted MIP nanoparticles was verified by their static water contact angles (Scheme 2b) and their stability and dispersibility in water (Scheme 3c). Finally, as can be seen in Scheme 2d, the successful incorporation of the fluorescent monomer was tested by observing its fluorescence emission under UV light (365 nm). The real and potential applicability of the nanoMIP sensor was demonstrated by separation (with recovery from 98% to 102%) and quenching quantification (with a LOD of 0.26 μM) of tetracycline in undiluted serums.

A fluorescent nanoMIP that determines nitroaromatic compounds by the reduction in fluorescence emission intensity (quenching) was also synthesized [70]. To achieve this goal, a fluorescent monomer (N-2-propenyl-(5-dimethylamino)-1-naphthalene sulphonamide) was added to the polymerization mixture where it non-covalently interacted with the templates (4-nitroaniline and 2,4-dinitroaniline). The nanoMIPs were prepared using a solid phase imprinting approach, and therefore, the templates were previously immobilized on activated glass beads. These selective sensors had short response times (around 11 min) and a high storage stability (after 100 days, fluorescence intensity had dropped less than 10%). A study of recovery was carried out in tap water, and the recovery was close to 100% for the two analytes. The LODs for 2,4-dinitroaniline and 4-nitroaniline were 6 and 7, respectively. Recently, an optical sensor based on fluorescence resonance energy transfer (FRET) in MIP nanoparticles has been prepared via three step-based approaches [71]. In this case, MIP nanoparticles containing orthogonal alkyne or azyde groups were firstly synthesized by precipitation polymerization in the presence of a therapeutic drug as the template (propranolol), then a Cu(I)-catalyzed 1,3-dipolar cycloaddition reaction was carried out between the alkyne or azide groups and an azide- or an alkyne-tagged organic amine. Finally, in the third step, an amine-reactive fluorescent dye was immobilized on the surface of the MIP nanoparticles. Once the template was removed from the MIP nanoparticles, the complete binding of the analyte in the sample solution (tap water) was achieved, and its concentration could be determined according to the fluorescence of the system.

Electrochemical sensors-based MIP nanoparticles are a great alternative to the use of systems where the fluorescence is the analytical signal, due to they are small and compact sensors, as well as fast, sensitive, and reproducible, allowing non-destructible sampling in situ. Therefore, a high number of works that involve the preparation and use of MIPs as chemical sensors have been published [72,73,74,75,76,77]. In this way, an electrochemical nanoMIP sensor, based on the initiated-radical copolymerization of MAA (functional monomer) and EGDMA (cross-linking monomer) in the presence of a template molecule (diazinon), has been reported [78]. The suspension polymerization was carried out at 65 °C for 24 h, and then the nanoMIP was mixed with graphite powder, and the mixture was packed into a Teflon tube to obtain a carbon paste electrode. Thus, the molar ratio of template/monomer, the electrode composition, the effect of washing time, and pH on the electrode response, as well as the most important electrochemical parameters, were deeply studied. Finally, the electrode, which showed good repeatability and stability with the time, was used to determine diazinon in well water and apple fruit samples obtaining satisfactory recovery values (92.53–100.86%). The LOD was 7.90 × 10^–10^ mol·L^−1^, which is a value comparable to other published methods for diazinon determination. A sensitive nanoMIP electrochemical impedance spectroscopy (EIS) sensor to determine cocaine in environmental samples was presented [79]. A scheme of the steps involved in the fabrication of the sensor is depicted in Scheme 3. In brief, 11-mercaptoundecanoic acid (MUA) was firstly anchored on the gold electrode, followed by the covalent attachment of the amine-functionalized nanoMIPs to the carboxyl groups of MUA. The sensor was able to bind cocaine with high detectability (LOD of 0.24 ng·mL^−1^) and selectivity even in the presence of morphine and levamisole (the most common cocaine cutting agent).

The same group has also been reported the fabrication of an α-casein nanoMIP sensor using surface plasmon resonance (SPR) [80]. In this case, 16-mercaptohexadecanoic acid (MHA) was immobilized to the surface of the gold SPR chip, and then it was linked to the nanoMIP. The sensor was able to the selective retention of α-casein in milk samples with a LOD of 127 ng·mL^−1^. On the other hand, Canfarotta et al. [81] have described, for the first time, the preparation of nanoMIP-based thermal sensors to determine several biomolecules. Thus, a wide range of templates chemically and structurally different (a small molecule, two peptides, and a protein) were imprinted independently (i.e., a MIP was synthesized for each template). The imprinting was carried out via a solid-phase approach on functionalized-glass beads. Next, nanoMIPs were placed on the surface of thermocouples in a liquid flow cell. In this way, when the analyte is retained on the nanoMIP, the heat-flow from the sensor to the liquid is blocked, and the temperature measured by the thermocouple is reduced, which is correlated with the concentration of the analyte in the sample solution. LODs were found in the low nanomolar range, which is a substantial improvement over microMIP-based thermal sensors previously reported.

### 4.2. Silica Nanoparticles

Silica is the most common core employed for the preparation of MIP, due to its advantages, such as thermostability, biocompatibility, great permeability, and stability under acid conditions [2]. Silica particles are easily synthesized by the Stöber method through the hydrolysis of tetraethoxysilane (TEOS) under a basic medium. Surface modification of the silica particles can be achieved by several silane coupling agents, such as 3-aminopropyltriethoxysilane (APTES), methacryloxypropyltrimethoxysilane (MPS), and vinyltriethoxysilane (VTES), which can easily conjugate with the silica particle surface through the active hydroxyl groups. These modification groups improve the reactivity of the silica surface to later receive the MIP shell.

Silica particles were employed for the preparation of a MIP for lysozyme recognition through a new synthesis strategy [82]. Non-specific recognizing sites were largely eliminated by the controlled addition of PEG to the MIP surface. Hydrophilic silica nanocores with template-capturing groups and high-density reversible addition-fragmentation chain transfer polymerization (AFCTP) were obtained employing click chemistry, which was covered by a MIP shell with lysozyme as a template. Before the template removal, PEG chains were attached to the molecularly imprinted shell to passivate non-specific recognizing sites. Physical parameters, such as length of the grafted PEG chains and thickness of the imprinted shells, were easily controlled through the polymerization time. The addition of PEG to the imprinted shell greatly improved the selectivity of the MIP compared to MIP without the addition of PEG, increasing the imprinting factor from 2.1 to 9.1. The new synthesis strategy was also employed for the imprinting of bovine hemoglobin, to prove the generality of the developed approach.

A new strategy to prepare molecularly imprinted silica particles employing APTES as a catalyst instead of the traditional basic catalysts was also presented [83]. APTES was also used as the functional monomer to prepare sites with high recognition ability of the target analyte. The use of APTES as the polymerization catalyst enhances the imprinting process by the elimination of detrimental effects caused by the use of additional catalysts, that can hinder the imprinting efficiency by disrupting the template-functional monomer complex in the pre-polymerization step. The new strategy was successfully employed to synthesize a new silica-based MIP for selective extraction of 1-naphthyl phosphate. The MIP was packed into an SPE cartridge and employed for the analyte extraction from water, achieving an enrichment factor of 40. A theoretical evaluation of the interactions between the template molecule and the functional monomer was carried out to provide more understanding of the recognition mechanisms. The new approach has the potential to be also employed in the preparation of high selective MIP for biomolecules, such as proteins and peptides.

Silica nanoparticles have also been explored for the imprinting of macromolecules, which has been increasing in the last decade, despite some limitations. The structural stability of the template is one of the most critical issues during the synthesis of macromolecules MIPs. In this sense, a new approach to minimize this inconvenience using macromolecular functional monomers for the imprinting of macromolecules, such as proteins, was proposed [84]. The structural stability of the template is preserved, due to the interaction of the macromolecularly functional monomers with the protein surface, instead of disturbing the hydrogen bonds that sustain the structural stability of the protein. The macromolecularly functional monomers were polymerized onto the surface of silica particles in the presence of the template protein, resulting in a MIP with an imprinting factor of 5.8, which was higher than obtained by the used of micromolecularly functional monomers that provided an imprinting factor of 3.4. Based on these observations, the use of silica nanoparticles as MIP supports is still an interesting approach, and combined with the use of macromolecularly functional monomers for the imprinting of proteins, has great potential to be explored on the protein imprinting, as they overcome an important drawback.

### 4.3. Carbon Nanoparticles

Carbon-based materials (CBMs) have been presented as a potential and advantageous alternative to silica nanoparticles in the field of analytical chemistry in applications involving MIPs, due to their capacity to bond to different elements and being chemically inert. Thus, the use of carbon-based materials (CBM) as a matrix provides high versatility on the selection of polymers and high robustness to the final sensor. Different types of CBM have been explored in the synthesis of MIPs, such as graphene oxide [85,86,87], carbon dots [88,89,90,91,92], and carbon nanotubes [93,94,95,96,97]. Most of these application employs CBM as a substrate to support the MIP, that can be synthesized in different shapes and sizes, depending on the selected CBM. The incorporation of CBM in MIPs also improves their performance by increasing the surface area and selectivity for the template molecules, due to the unique properties of the CBM. Additionally, to these improvements, the incorporation of CBM in MIPs provides the ability to use these MIP materials for electrochemical sensing, due to the conductive properties of the CBM [12].

**Scheme 4:**
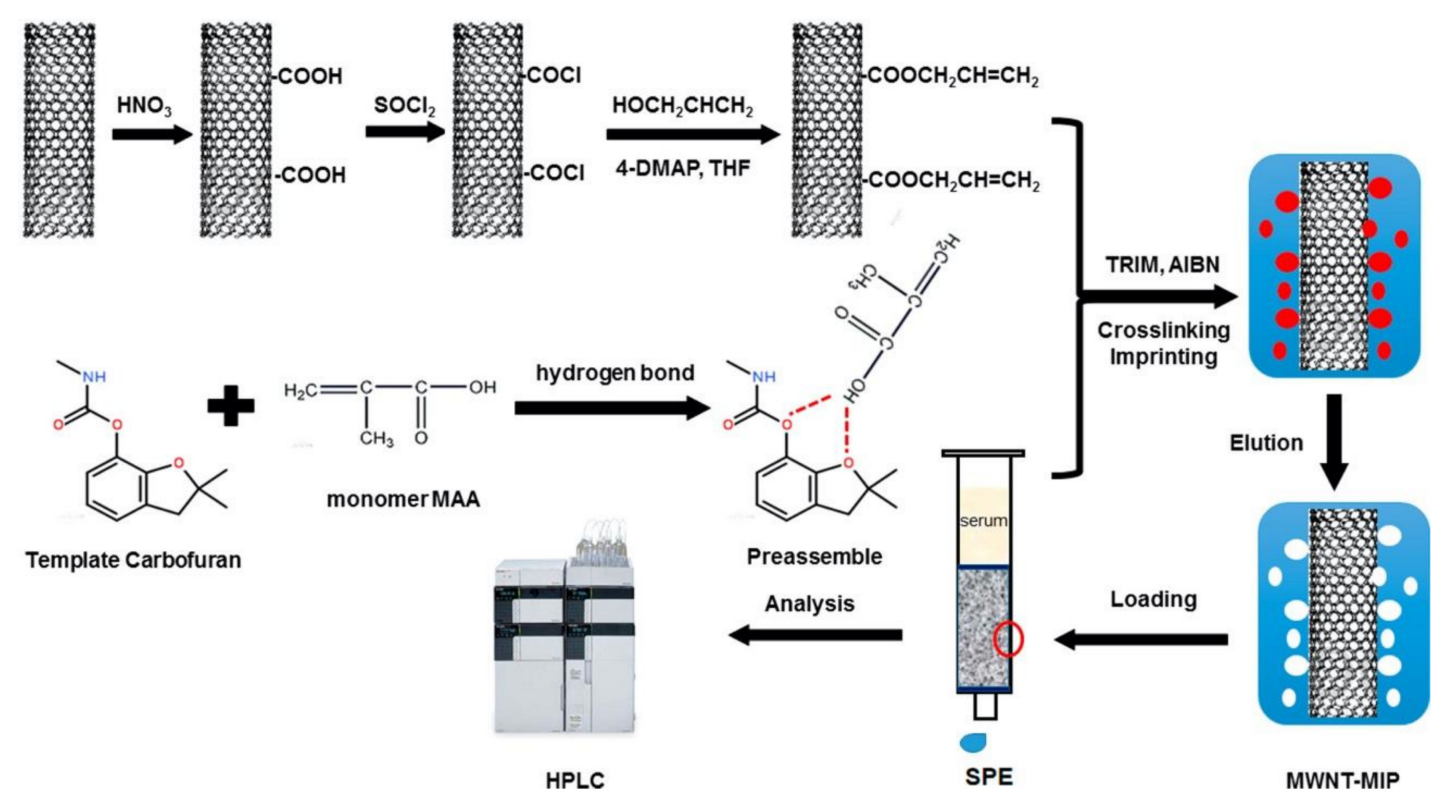
Synthetic scheme for multi-walled carbon nanotube-MIP and application as an absorbent for SPE of Carbofuran. Reproduced with permission from [98].

A new MIP based on multi-walled carbon nanotubes (MWCNTs) for the selective extraction of carbofuran, a carbamate pesticide, was presented [98]. A thin layer of the MIP was synthesized onto the surface of MWCNTs by the polymerization of methacrylic acid, as functional monomer and trimethylolpropane trimethacrylate as a cross-linking agent and carbofuran as a template. The characterization of the final material confirmed the effective grafting of the thin layer of MIP onto the MWCNTs surface. The material was employed for the solid-phase extraction of carbofuran from human serum and analyzed by high-performance liquid chromatography. A general scheme of the synthesis and application of the MIP supported onto MWCNTs surface is shown in Scheme 4. Carbofuran recovery was in the range of 89% to 94%, indicating a high recognition ability of this material.

Graphene oxide was employed to synthesize a double-sided magnetic molecularly imprinted polymer for selective recognition of microcystins [87]. First, Fe_3_O_4_ magnetic nanoparticles were coated by the MIP constituted by diphenylethene and acrylamide. These particles were later anchored into both sides of the GO sheets, resulting in a double-sided imprinted material, that was employed for the preparation of a magnetic solid-phase extraction procedure for the selective extraction and preconcentration of traces of eight microcystins in water samples for the later separation and detection by LC-MS/MS. An enrichment factor of 2000, limits of quantification in the range of 0.1 to 2.0 ng·L^−1^, and a recovery ranging from 84% to 98% was achieved after optimization of the extraction parameters. These analytical features are superior to those observed for procedures previously presented for the same application, which highlights the contribution of the MIP for the improvement of the analytical procedure. Additionally, to the analytical capacity, the new material has a great potential for the removal of microcystins from environmental waters.

Carbon dots are a new class of carbonaceous nanoparticles in the range of 1 to 10 nm with unique tunable photoluminescence properties [99]. They are presented as a potential alternative to quantum dots (QDs), since they offer improved biocompatibility and low toxicity, enhancing their potential in applications, such as bio-diagnosis, sensors, and bio-imaging [100,101]. Carbon dots (CDs) are usually combined with different solid matrix or synthesized in solid-state nanocomposites to improve their stability and performance [102]. Additionally, the use of these solid matrix provides greater versatility to CDs applications by controlling the shape, size, interparticle distances, porosity, and variable mechanical strength of the final material. The great photoluminescence properties can be exploited in several fields ranging from drug delivery to bioimaging; however, the CDs present poor selectivity, which can limit their applications. In this sense, the combination of CDs with MIPs is a suitable strategy to improve the fluorescence selectivity of CDs, resulting in materials with great physical and chemical stability and reusability.

A new approach to synthesize carbon dots embedded metal-organic framework at MIP (CDs@MOF@MIP) nanoparticles for the sensitive and selective determination of quercetin was presented [91]. The synthesis of the metal-organic frameworks was performed through a room temperature reaction and later employed to produce the sensor. CDs with high blue luminescence were added as a signal transducer, which produces a detectable fluorescence signal by the sensing of the interactions between the analyte and the molecularly imprinted polymer. Additionally, to the high detectability provided by the fluorescence emission and the high selectivity provided by the molecular imprinting process, the CDs@MOF@MIP sensor presented a faster reaction rate than a sensor prepared with CDs-embedded molecularly imprinted polymer in the absence of the metal-organic framework. The CDs@MOF@MIP presented a limit of detection of 2.9 nM with an RSD of 1.9%. It was employed to determine quercetin in Ginkgo biloba extract capsules, and the results were statically comparable to those obtained by high-performance liquid chromatography.

The incorporation of CBM in MIPs has also been explored to the fabrication of new sensors to determine different analytes in biological samples, HIV-p24 virus [93], testosterone [103], insulin [104], and melatonin [105]. A review exploring the application of carbon materials to the preparation of MIP for application in biological samples was recently published, and detailly presents the most important advances in the field [12]. A simple, fast, and sensitive procedure to determine HIV-p24 based on MIP synthesized onto the surface of an MWCNTs modified glassy carbon electrode was presented [93]. The polymer was obtained through the polymerization of acrylamide, as the functional monomer, N, N′-methylenebisacrylamide as the cross-linker, and ammonium persulphate as the reaction initiator. The obtained sensor presented improved performance compared to most HIV-p24 available methods, with a limit of detection of 0.083 pg cm^−3^. It was successfully applied to the determination of the virus in human serum samples, achieving satisfactory reliability and accuracy confirmed by comparison with enzyme-linked immunosorbent assay (ELISA).

### 4.4. Gold Nanoparticles

Gold nanoparticles (AuNPs) are usually employed as a support matrix for MIP, as well as the carbon and magnetic nanoparticles. However, AuNPs also improve the analytical signal when MIPs are used as electrochemical sensors, due to their direct and fast electron transfer between a substrate and a broad series of molecules, which turn them into an appealing alternative for the amplification of electrochemical signals [106,107].

**Scheme 5:**
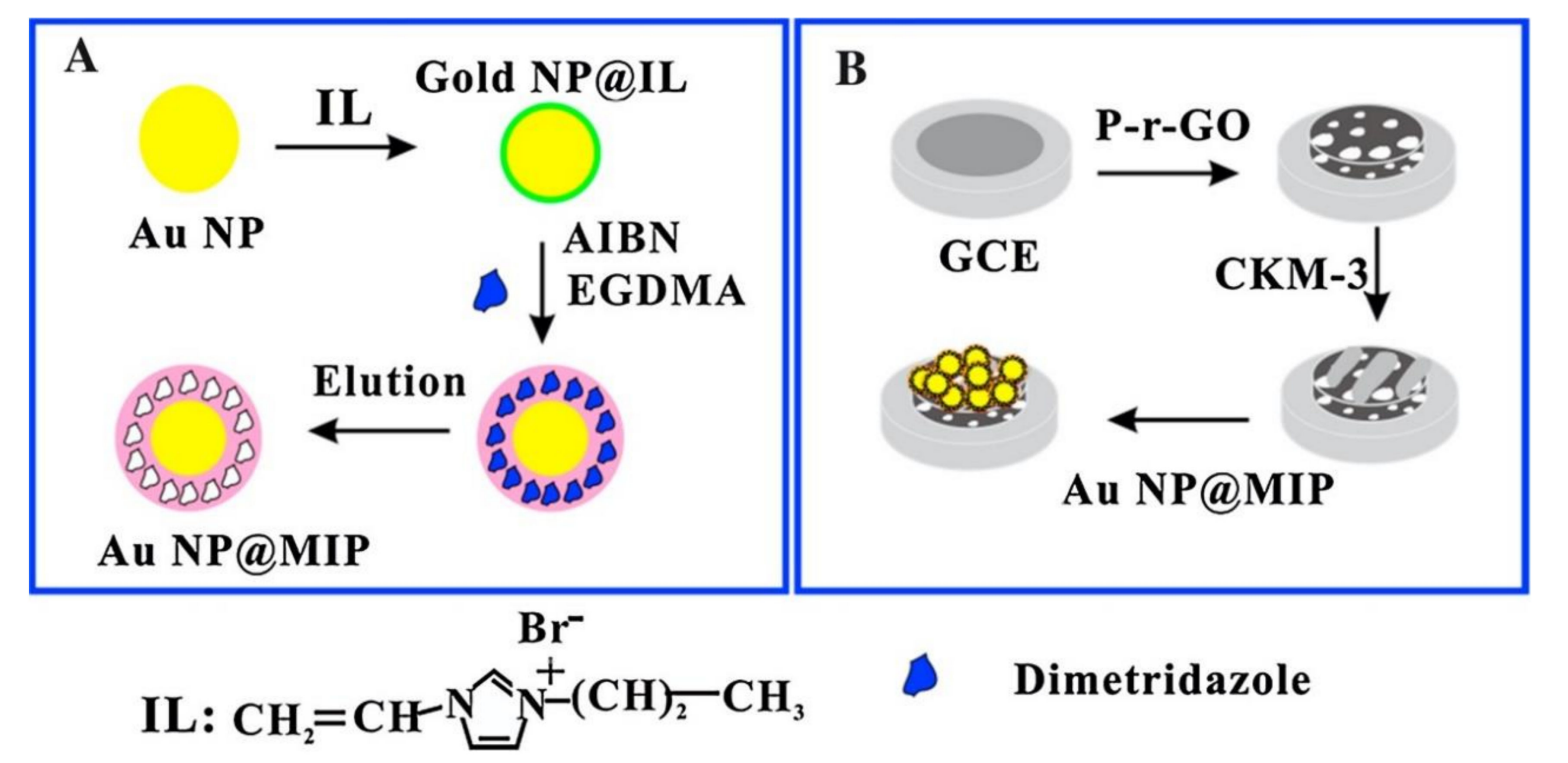
Schematic diagram of the construction of the (**A**) molecularly imprinted gold nanoparticles and (**B**) their use on the glassy carbon electrodes coating. Reproduced with permission from [106].

A new and easy procedure to prepare MIPs supported on AuNPs, which were later used for the coating of porous graphene and mesoporous carbon modified glassy carbon electrode for the selective determination of dimetridazole was reported [106]. AuNPs were obtained by the reduction of HAuCl_4_, which were coated by 3-propyl-1-vinylimidazolium bromide, an ionic liquid that was employed as a monomer to synthesize the MIP. The molecularly imprinted AuNPs were later coated on the modified glassy carbon electrodes, which presented a selective and sensitive response to dimetridazole with a limit of detection of 5.0 × 10^−10^ mol·L^−1^. The applicability of the new sensor was proved by the determination of dimetridazole in food samples. A scheme demonstrating the synthesis of the sensor is presented in Scheme 5.

A new water-compatible MIP sensor decorated with AuNPs by the combination of molecular imprinting and macromolecular self-assembly for the selective determination of glucose was presented [108]. An amphiphilic copolymer containing imino groups was obtained through radical copolymerization, which was later interacting with the template molecules, resulting in nanoparticles embedded with glucose. AuCl^4−^ was added as a precursor to generating in situ AuNPs by the reduction action of the protonated imino groups from the copolymer. The amount of AuNPs generated was easily controlled by adjusting the pH value of the reactional mixture. The final MIP particles were integrated onto the surface of a gold slide by electrodeposition, forming a MIP film that was cross-linked by UV irradiation. The AuNPs formed in the MIP significantly improved the conductivity of the film by the direct electric contact between the gold surface and the recognition sites of molecular cavities of the imprinted polymer, resulting in enhanced detectability. Glucose was detected with a limit of detection of 3 × 10^−12^ mol·L^−1^. These analytical features were better than those obtained by the same sensor in the absence of the AuNPs, which highlights their enhancing effect.

Hybrid molecularly imprinted AuNPs were reported for the selective recognition of ciprofloxacin [109]. The chitosan-based AuNPs were synthesized by adding chitosan flakes during the AuNP production. The hybrid nanoparticles were employed to support a MIP that was obtained by the polymerization of methacrylic acid as the functional monomer and ethylene glycol dimethacrylate as the cross-linker agent. The final MIP was used to modify the surface of a glassy carbon electrode that provided great detectability, achieving a limit of detection of 210 nmol·L^−1^. The enhanced selectivity and sensitivity are related to the MIP and the hybrid AuNPs, respectively. The applicability of the MIP sensor was proved by its application to the determination of ciprofloxacin in different samples, such as milk, mineral water, tap water, and pharmaceutical formulations with recovery values in the range of 94% to 106%.

AuNPs were also employed to improve the electron-transfer rate of a new MIP for the selective electrochemical determination of patulin. The sensor was prepared by the polymerization of aminothiophenol as a functional monomer and 2-oxindole as a template molecule. AuNPs, chitosan, and carbon dots were incorporated into the MIP to enhance the electrochemical performance of the sensor that was used to modify the surface of a glassy carbon electrode. The final sensor presented enhanced performance, achieving a limit of detection of 7.57 × 10^−13^ mol·L^−1^ of patulin with a fast signal response, high selectivity, and suitable stability [110].

### 4.5. Magnetic Nanoparticles

The use of magnetic nanomaterials for extraction and preconcentration of different analytes from complex matrices has been extensively explored in the last decades [111]. Advantages, such as the easy separation of the MNPs from the sample solution by applying an external magnetic field and the possibility of functionalization with different functional groups, were responsible for the great popularity of the magnetic solid-phase extractions (MSPE) [112]. The selectivity of MSPE was later enhanced using MIP as coatings of the magnetic core, which resulted in materials that combine the advantages of the MNPs with the high selectivity provided by the MIP.

The synthesis of magnetic molecularly imprinted polymers (MMIPs) usually comprises of four steps: (i) The synthesis of the magnetic core, such as Fe_3_O_4_; (ii) the surface functionalization or modification of the MNPs; (iii) the polymerization of the MIP onto the surface of the functionalized magnetic cores in the presence of a template analyte, cross-linker agent and functional monomer [113]. The choice of the coating material for the MNPs influences the final chemical stability of the MMIP and could limit its applications to a wide range of samples. Silica-based coatings present significant instability of the siloxane bonds under acid and basic media. However, this limitation can be overcome by using polymeric shells that are resistant to solutions with low and high pH values. Additionally, polymeric shells can be easily modified and grafted, which is a desirable advantage to prepare MIPs. Since the introduction of MMIPs, several approaches have been proposed for different analytes, such as food dyes [114], vitamin D [115], diethylstilbestrol, hexestrol, and dienestrol and bisphenol A [116,117], citrinin [113], and lysozyme [118].

**Scheme 6:**
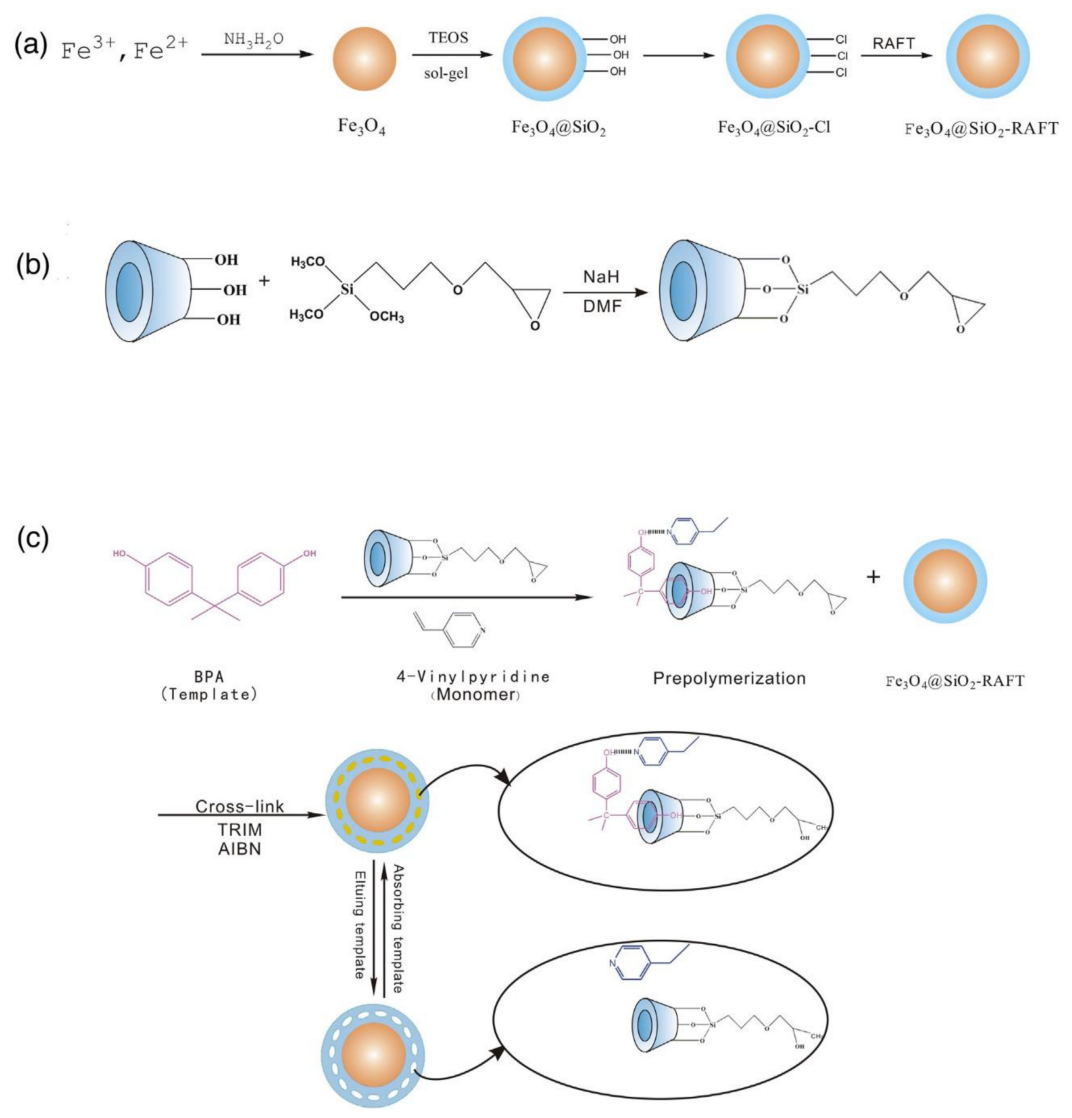
Synthesis route of surface-imprinted core-shell magnetic beads and their application for extraction of bisphenol A. (**a**) Fe_3_O_4_ magnetite particles synthesis by a co-precipitation method followed by silica coating and functionalization with chloromethyl-phenyl groups; (**b**) β- cyclodextrin modified with an epoxy bond; (**c**) 4-vinylpyridine and β- cyclodextrin used as binary functional monomers for the synthesis of the MIP for bisphenol A. Reprinted with permission from [119].

A novel strategy for extraction and preconcentration of bisphenol A from milk employing a MIP with MNPs core synthesized through a reversible AFCTP was reported [119]. Β-cyclodextrin and 4-vinyl pyridine were employed as functional monomers and bisphenol A as the template molecule. Before polymerization, bisphenol A forms an inclusion complex with β-cyclodextrin, which interacts with 4-vinyl pyridine through hydrogen interactions. Thereby, highly selective cavities in the final MMIP are achieved, which was proved by its superior selectivity for bisphenol A compared to the NIP. The general synthesis scheme is shown in Scheme 6. The MMIP also provided improved detectability achieving a limit of detection of 3.7 µg·L^−1^ and adequate accuracy and absence of matrix effects demonstrated by the recovery from 97% to 99%.

A carbon QDs-doped MMIP for selective fluorescence detection of N-acyl homoserine lactones was presented [120]. The magnetic core was composed of Fe_3_O_4_ core covered by a MIP. The MIP was obtained by the polymerization of methacrylic acid and HEMA as functional monomers, ethylene glycol dimethacrylate as a cross-linking agent, and 2,2′-azobis(2-methylpropionitrile) as the reaction initiator. The combination of MNPs and carbon QDs at the same MIP resulted in a final material with superparamagnetism, fluorescence, high selectivity, and fast response properties. The MMIP particles were employed as the recognition unit of the sensor for N-acyl homoserine lactones, that quenched the fluorescence of the probe in a range of concentration from 3.65 nmol·L^−1^ to 960 nmol·L^−1^. The sensor was employed to determine the N-acyl homoserine lactones in complex samples, such as fish juice and milk, with satisfactory recoveries in the range from 83% to 90% and RSD lower than 5.1%. The analytical performance of the MMIP combined with carbon QDs highlighted the potential of magnetic fluorescence probes for a wide range of applications in different types of samples.

## 5. Conclusions

Several advances have been made in the field of MIP technology in the last decades. Different approaches and techniques have been presented to synthesize MIP with different sizes, shapes, desirable chemical/physical properties, and particle size distribution. Nanomaterials have an important role in this scenario, as they are crucial tools to achieve these improvements. In this review, we focused on the contribution of several nanomaterials to the progress of MIP technology. They have been employed to modulate size, detectability, and shape of the imprinted materials, and their use depends on the chosen application. Silica nanomaterials have been exploited mainly as matrix support for MIP, due to their easy functionalization and different shapes, and to bioanalysis, due to their biocompatibility. Other nanomaterials have been employed mainly for the enhancement of detectability, such as carbon-based materials and AuNPs that are great approaches to improve the response of sensors based on MIP, especially for electrochemical determinations. The preparation of MIP for small molecules is already consolidated and was extensively explored in the last decades. Most of the current imprinted materials are focused on developing new approaches for the selective and sensitive recognition of macromolecules and large analytes, such as proteins, peptides, and viruses. The advances in the synthesis of new nanomaterials and the improvement of the physical/chemical properties of the current nanomaterials must bring impressive advances to the MIP field in the next years, allowing their application to more complex analytical problems. Despite all the improvements provided by the use of different micro and nanomaterials, some limitations on the imprinting process still remain challenging, mainly by the nature of the template and the formation of non-specific binding sites, which hinder selectivity and detectability. Futures trends in this field may include the exploitation of multi-hybrid micro and nanocomposites composed by AuNPs-MIPs and some other kind of nanomaterial, such as metals, oxides, or carbon-based—which could be suitable alternatives to improve the physical properties of the MIPs.

## Abbreviations

AIBN	azobisisobutyronitril
AFCTP	addition-fragmentation chain transfer polymerization
AM	acrylamide
AnHEMA	2-hydroxyethylanthrancene-9-carboxylate)methacrylate
APTES	3-aminopropyltriethoxysilane
AuNP	gold nanoparticle
CA	caffeic acid
CBM,	carbon-based material
CBZ	carbendazim
CD	carbon dot
CDs@MOF@MIP	carbon dots embedded metal-organic framework at MIP
ChCl	choline chloride
DES	deep eutectic solvent
DMC	5,7-dimethoxycoumarin
EGDMA	ethylene glycol dimethylacrylate
EG	Ethylene glycol
EIS	electrochemical impedance spectroscopy
ELISA	enzyme-linked immunosorbent assay
FLD	fluorescence detector
FRET	fluorescence resonance energy transfer
GO	graphene oxide
h-BN	hexagonal boron nitride
HEMA	2-hydroxyethyl methacrylate
HPLC	high performance liquid chromatography
LC-MS/MS	high-performance liquid chromatography-tandem mass spectrometry
IF	imprinting factor
LIF	laser-induced fluorescence detection
LOD	limit of detection
MAA	methacrylic acid
MIM	molecularly-imprinted monolith
MMIP	magnetic molecularly imprinted polymers
MNP	magnetic nanoparticle
MWCNT	multi-walled carbon nanotube
MIP	molecularly imprinted polymer
MPS	methacryloxypropyltrimethoxysilane
MS	mass spectrometry
MSPE	magnetic solid-phase extractions
MHA	16-mercaptohexadecanoic acid
MUA	11-mercaptoundecanoic acid
NIP	molecularly non-imprinted polymer
POSS	polyhedral oligomeric silsesquioxanes
QD	quantum dot
RSD	relative standard deviation
SPE	solid phase extraction
SPR	surface plasmon resonance
TBZ	thiabendazole
TEOS	tetraethoxysilane
UHPLC	ultrahigh-pressure liquid chromatography
UV	ultraviolet
VTES	vinyltriethoxysilane
4-VP	4-vinylpyridine
